# Relevance of control diet choice in metabolic studies: impact in glucose homeostasis and vascular function

**DOI:** 10.1038/s41598-020-59674-0

**Published:** 2020-02-19

**Authors:** Raquel González-Blázquez, Martín Alcalá, María S. Fernández-Alfonso, Palmira Villa-Valverde, Marta Viana, Marta Gil-Ortega, Beatriz Somoza

**Affiliations:** 1 Departamento de Ciencias Farmacéuticas y de la Salud, Facultad de Farmacia, Universidad San Pablo-CEU, CEU Universities, 28925 Madrid, Spain; 2 Departamento de Química y Bioquímica, Facultad de Farmacia, Universidad San Pablo-CEU. CEU Universities, 28925 Madrid, Spain; 3 Instituto Pluridisciplinar, Unidad de Cartografía Cerebral, Universidad Complutense de Madrid, 28040 Madrid, Spain; 4 Departamento de Farmacología, Facultad de Farmacia, Universidad Complutense de Madrid, 28040 Madrid, Spain

**Keywords:** Peripheral vascular disease, Pre-diabetes, Risk factors

## Abstract

The experimental approach for the study of cardiometabolic disorders requires the use of animal models fed with commercial diets whose composition differs notably, even between diets used for control groups. While chow diets are usually made of agricultural by-products, purified low-fat diets (LF) contain a higher percentage of easy metabolizable carbohydrates, together with a reduced amount of polyunsaturated fatty acids, micronutrients and fiber, all associated with metabolic and vascular dysfunction. We hypothesize that the LF diet, commonly used in control animals, could promote adverse vascular and metabolic outcomes. To address this issue, 5-week-old male C57BL6J mice were fed with a standard (Chow) or a LF diet for 6 weeks. Changes in body weight, adiposity, biochemical parameters, systemic and aortic insulin sensitivity and endothelial function were recorded. LF diet did not modify body weight but significantly impaired systemic glucose tolerance and increased triglycerides and cholesterol levels. Endothelial function and aortic insulin sensitivity were significantly impaired in the LF group, due to a reduction of NO availability. These findings highlight the importance of selecting the proper control diet in metabolic studies. It may also suggest that some cardiometabolic alterations obtained in experimental studies using LF as a control diet may be underestimated.

## Introduction

It is currently well known that high fat (HFD) and/or high sugar (HSD) diets are irrefutably associated with metabolic and cardiovascular (CV) risk. Indeed, a link between hypercaloric diets and chronic diseases such as hypertension, insulin resistance and dyslipidemia and their associated complications have been reported^1^. In this regard, it has been shown that the intake of high quantities of sucrose is associated with the development of type 2 diabetes and CV disease^2^. Similarly, several studies of our group have shown that the intake of HFD accounts for the development of vascular endothelial dysfunction correlating with an increase in plasmatic levels of non-esterified fatty acids (NEFA), triglycerides (TG) as well as an impaired glucose management^3–5^.

In the design of an experimental animal model to approach the study of these conditions *in vivo*, selecting the proper diets becomes a key step that would potentially define the outcome and the relevance of the data obtained. Nowadays, there is a wide variety of both commercial and hand-made diets available to induce metabolic disturbances or vascular damage. Traditionally, the diet of choice was selected due to quantitative reasons, attending to the elevated content in carbohydrates or fat that would best mimic the condition under investigation. However, the qualitative composition has gained interest over the last years and now the focus is set in the origin and characteristics of the macronutrients. For instance, it has been described that while carbohydrates sources in chow diets are mainly ground wheat, corn, or oats, alfalfa and soybean meals (with a major content of complex carbohydrates), the main source in purified diets are sucrose, dextrin and maltodextrose, all of them easily-metabolizable sugars that may have play a negative role in metabolic and cardiovascular diseases. Furthermore, while chow diets contain a wide variety of fiber sources (both soluble and non-soluble), purified diets only contain cellulose, a non-soluble type of fiber^6^.

A similar differential composition between chow and purified diets is observed regarding fat content, especially in terms of fatty acids. In this regard, while fat contained in chow diets is mainly supplied by a variety of vegetable and fish oil sources, purified diets contain mainly fat from lard, significantly richer in saturated fatty acids and with a very limited amount of polyunsaturated fatty acids (PUFA)^6^. In this direction, it has been suggested that saturated fatty acids are associated with vascular alterations whereas PUFA (mainly linoleic and linolenic acid) improve endothelium dependent relaxation and insulin sensitivity^7^. Finally, a differential supplementation with micronutrients like vitamins or minerals is also a typical feature when comparing the composition of experimental diets^6^.

Usually, the selection of the experimental diets that are used in the intervention groups (ie, HFD in obesity studies) is a thorough decision based on the characteristics of the study. However, the selection of the control diet could become an automatic process, but due to the aforementioned differences between chow and purified diets, the use of one or another may also impact in the biology of the control animals, resulting in an under or overestimation of the obtained results.

Since it has been suggested that not only the energy provided by a diet but also the nature of nutrients might be crucial for the development of both metabolic and vascular alterations, we hypothesize that the differential composition of the chow and low fat (LF) purified diet in terms of carbohydrates, lipids and micronutrients, even in diets that are normocaloric might impair glucose tolerance and endothelial function. Therefore, the aim of this study was to assess the effect of a LF diet in comparison with a standard chow diet for 6 weeks on: i) body weight and adiposity, ii) biochemical parameters, iii) systemic insulin sensitivity, iii) endothelial function, and iv) vascular insulin sensitivity.

## Results

### LF diet provides a higher amount of easily metabolizable carbohydrates and cholesterol but lower quantities of soluble fiber and polyunsaturated fatty acids than the Chow diet

Chow and LF diets are considered isoenergetic. However, important nutritional differences were found when we analyzed the composition of both diets using data provided by the manufacturers (Table 1). First, chow diet has a lower carbohydrate content (44.2%), obtained from agricultural byproducts, while LF content is not only higher (67.4%), but also obtained from easily metabolizable sources, such as sucrose, dextrin and maltodextrin. The second main difference is related to the fiber content and origin. While the net content of crude fiber is lower in the chow than in the LF diet (3.5 *vs* 4.7), chow diet contains several types of fiber (soluble and non-soluble) and not only cellulose like the LF diet (Table 1). Finally, although the lipid content is similar between the two diets, chow diet is richer in ω-3 and ω-6 polyunsaturated fatty acids, while LF diet has a higher cholesterol content in addition to a reduced fat-soluble vitamin content. Similarly, NMR (nuclear magnetic resonance) analyses revealed a higher percentage of total sugars in the LF diet as compared with the chow diet (Chow: 43.4 vs LF: 76.8%). In addition, we observed a significant increase in the amount of easy metabolizable sugars like maltotriose, glucose and sucrose in the LF diet (Chow: 0.03 vs LF: 0.76).

**Table 1:** Composition and energetic profile of standard chow (Chow) and low-fat (LF) diet.

	Chow	LF
Energetic profile		
Total energy (Kcal/g)	3.1	3.76
Total calories provided by protein (%)	24	18
Total calories provided by carbohydrates (%)	58	71.8
Total calories provided by fat (%)	18	10.2
Total protein (%)	18.6	16.9
Casein (%)	—	18.9
Total carbohydrates (%)	44.2	67.4
Carbohydrates source	Ground wheat	Sucrose (33.12%)
	Ground corn	Dextrin (29.85%)
	Wheat middlings	Maltodextrin (3.32%)
	Dehulled soybean meal	
	Corn gluten	
Crude Fiber (%)	3.5	4.7
Neutral detergent fiber (%)	14.7	—
Fiber source	Cellulose	Cellulose
	Hemicellulose	
	Lignin	
Total fat (%)	6.2	4.3
Cholesterol (ppm)	—	18
Total saturated fatty acid (%)	0.9	1.14
C16:0 Palmitic (%)	0.7	—
C18:0 Stearic (%)	0.2	—
Total monounsaturated fatty acid (%)	1.3	1.3
C18:1; w-9 Oleic (%)	1.2	—
Total polyunsaturated fatty acid (%)	3.4	1.59
Linoleic acid (C18:2; ω-6) (%)	3.1	1.39
Linolenic acid (C18:3; ω-3) (%)	0.3	0.19
Fat source	Soybean oil	Soybean oil (2.37%)
		Lard (1.9%)
Vitamin A (IU/g)	15	3.8
Vitamin D-3 (IU/g)	1.5	0.9
Vitamin E (IU/Kg)	110	49.3

Nutritional composition is expressed in % (W/W).

### Body weight and tissues size were similar in animals fed with chow and LF diets except for SCAT (subcutaneous adipose tissue)

As shown in Table 2, body weight (BW) was similar in both Chow and LF animals after 6-weeks of diet. Although the average food intake was similar between the two groups (Chow = 3.0 ± 0.06 *vs* LF = 3.0 ± 0.03 g/day/mice), the average kcal consumption was significantly higher in the LF compared with Chow mice. Nevertheless, caloric efficiency was similar in both groups (Chow = 0.015 ± 0.0003 *vs* LF = 0.014 ± 0.0002 g/Kcal). When we analyzed the weight of several organs and the amount of fat in several adipose depots, we did not find differences between groups in the liver, the heart, the amount of visceral adipose tissue [perirenal adipose tissue (PR-AT) and mesenteric AT (Mes-AT)] and the amount of periaortic AT (PA-AT). Intriguingly, subcutaneous (SC-AT) weight was higher in Chow than in LF animals (30.75%). Animal growth was also similar in both groups as assessed by tibia length (Chow: 22 ± 0.0 cm *vs* LF: 22 ± 0.2 cm).

**Table 2:** Effect of dietary treatment on body weight, adiposity and biochemical parameters after 12 h fasting.

	Chow	LF
Body weight (g)	27.0 ± 0.5	27.2 ± 0.4
Energy intake (kcal/day/mice)	9.4 ± 0.2	11.5 ± 0.4*
PR-AT (mg/mm)	3.2 ± 0.3	3.0 ± 0.5
Mes-AT (mg/mm)	5.5 ± 0.5	5.8 ± 0.6
PAT-AT (mg/mm)	1.4 ± 0.2	1.6 ± 0. 2
SC-AT (mg/mm)	9.7 ± 0.3	7.4 ± 0.5**
Liver (mg/mm)	59 ± 2	54 ± 2
Heart (mg/mm)	6 ± 0.1	6 ± 0.3
Fasting parameters:		
Plasma glucose (mg/dL)	98.7 ± 0.8	108.0 ± 11.9
Plasma insulin (μg/L)	0.2 ± 0.03	0.6 ± 0.1*
Plasma TG (mg/dL)	43.1 ± 6.4	121.2 ± 15.4*
Plasma cholesterol (mg/dL)	65.1 ± 0.4	85.6 ± 5.6*
Plasma NEFA (mM)	1.2 ± 0.1	0.7 ± 0.2*
HOMA-IR	0.24 ± 0.1	1.2 ± 0.2*

Tissue weights are expressed in mg per mm tibia length, which was not different between groups. *P < 0.05, **P < 0.01 *vs* group Chow diet (Student’s *t*-test). Data are expressed as mean ± SEM of 5–10 determinations per group. Perirenal (PR-AT), mesenteric (Mes-AT), periaortic (PA-AT) and subcutaneous adipose tissue (SC-AT). Triglycerides (TG), non-esterified fatty acid (NEFA).

### LF diet impairs glucose tolerance and lipid metabolism

As summarized in Table 2, no differences were observed in circulating fasted glucose. However, glucose homeostasis was maintained due to a moderate but significant increase in insulin levels in LF animals compared to the Chow group. Accordingly, HOMA-IR index was also higher in LF mice than in the Chow group. Regarding the quantification of plasma lipids, fasting triglycerides (TG) and cholesterol were increased in LF mice, while NEFA were decreased.

**Figure 1:**
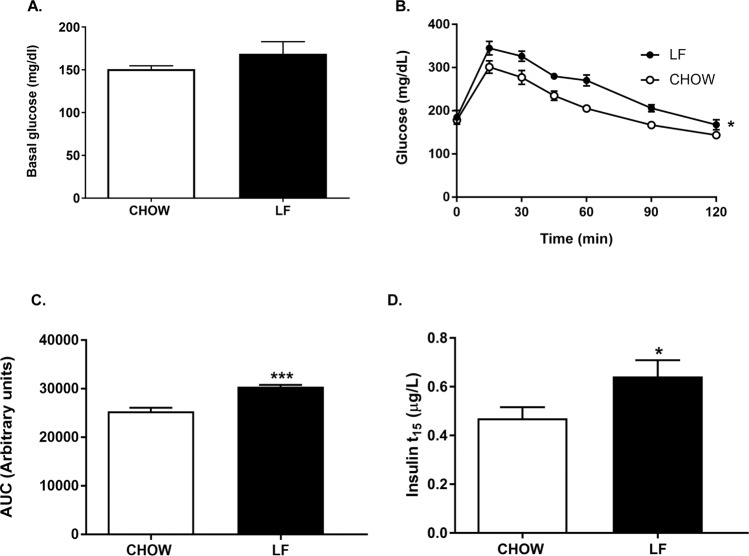
(**A**) Diagram bars show basal glucose levels before glucose administration. (**B**) Glucose concentrations during GTT, (**C**) AUC during the GTT in Chow and LF mice and (**D**) insulin levels assessed 15 min after glucose administration. Data are expressed as mean ± SEM of 7–10 determinations per group. *P < 0.05 compared with Chow mice, Student’s *t*-test (panels A,C); Two-way analysis of variance (ANOVA) followed by Bonferroni’s comparison test (panel B).

To get a further insight into the metabolic phenotype induced by both diets, we performed a glucose tolerance test (GTT) one week before sacrifice in mice fasted for 6 h. 30 minutes before the beginning of the test, basal glucose was measured. No differences were observed between the 2 groups (Fig. 1A). As it is illustrated in Fig. 1B, an ip. glucose bolus of 1 g/kg induced, 15 min later, a maximal increase in glycaemia in both experimental groups that was significantly higher in LF animals. While Chow mice recovered their basal glucose levels 90 min after glucose administration, LF mice needed 30 more min to reach basal levels (Fig. 1B). Consequently, the AUC showed a slightly worse management of glucose overload in mice fed with LF diet than in Chow group (Fig. 1C). Furthermore, plasma insulin levels measured 15 min after glucose administration were significantly higher in LF than in Chow mice (Fig. 1D).

### LF diet decreased vascular contractile responses to phenylephrine and reduced NO bioavailability

**Figure 2:**
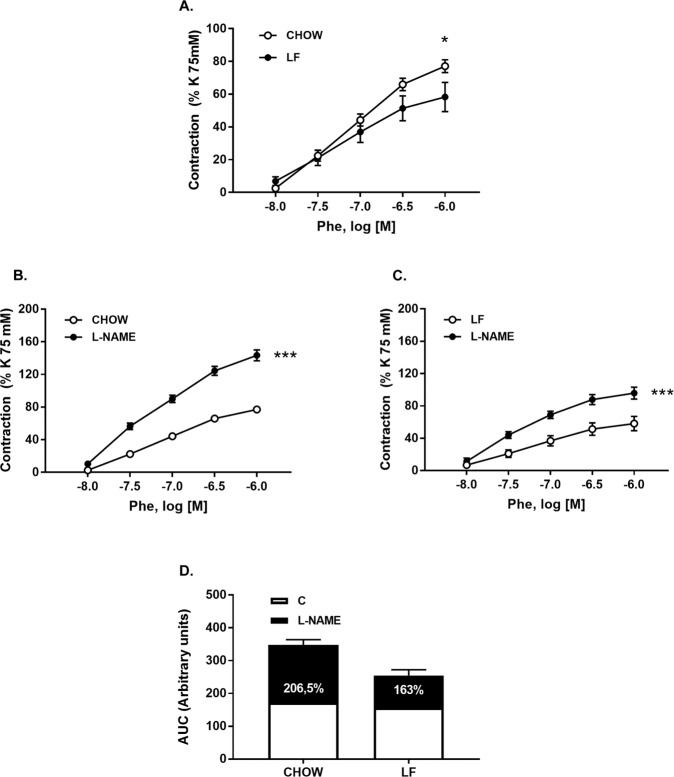
(**A**) Cumulative concentration-response curves to phenylephrine (10^−8^–10^−6 ^M) in aortic segments from both Chow and LF animals. (**B**,**C**) Cumulative concentration-response curves to phenylephrine (10^−8^–10^−6^ M) in aortic segments from both Chow (**B**) and LF (**C**) animals pre-incubated or not with L-NAME (10^−4 ^M). (**D**) Bar diagrams showing AUC from cumulative concentration-response curves to phenylephrine (10^−8^–10^−6 ^M) in aortic segments in presence or not of L-NAME. The percentage of increased contractile responses elicited by L-NAME and shown in black bars indirectly reflects basal NO availability. Data are expressed as mean ± SEM of 7–10 determinations per group. *P < 0.05 and ***P < 0.001 compared with the corresponding control group, Two-way analysis of variance (ANOVA) followed by Bonferroni’s comparison test (panels A–C), Student’s *t*-test (panel D).

Vascular contractility was analyzed in the thoracic aorta. No differences were observed between groups in the maximal contractile capacity assessed by contractions to 75 mM KCl (Chow = 0.73 ± 0.03 g *vs* LF = 0.64 ± 0.06 g). However, cumulative doses of phenylephrine (Phe; 10^−8^–10^−6^ M; Fig. 2A) elicited a significantly higher contraction in arteries from Chow animals affecting both the maximal response (E_max_) and the potency (pD_2_) (Table 3) compared with the LF mice.

**Table 3:** Maximal responses (E_max_) and potency (pD_2_) from concentration-response curves.

	Chow		LF	
	E_max_	pD_2_	E_max_	pD_2_
Phe	76.4 ± 3.8	6.9 ± 0.1	58.3 ± 8.9*	7.1 ± 0.1*
Phe + L-NAME	143.5 ± 6.7^###^	7.2 ± 0.02^###^	96.0 ± 7.4^##^	7.3 ± 0.1
Ach	87.6 ± 0.9	7.1 ± 0.04	80.3 ± 1.3***	6.6 ± 0.1***
Ach + L-NAME	12.4 ± 0.9^###^	6.5 ± 0.3^#^	10.4 ± 1.8^###^	7.2 ± 0.6^#^
Ach + INDO	68.1 ± 4.0^###^	6.4 ± 0.1^###^	63.2 ± 4.5^###^	6.3 ± 0.1
Insulin	59.3 ± 1.1	7.4 ± 0.1	48.0 ± 1.5***	7.1 ± 0.2*
Insulin + L-NAME	13.9 ± 1.7^###^	7.2 ± 0.2	19.9 ± 3.4^###^	7.8 ± 0.3

E_max_ is the maximal response to Phe, Ach or Insulin; pD_2_ is the negative logarithm of molar concentration of Phe, Ach or Insulin causing half maximal responses. Data are expressed as mean ± SEM of 7–10 determinations per group. *P < 0.05, **P < 0.01 and ***P < 0.001 compared with the Chow group. ^#^*P* < 0.05, ^##^*P* < 0.01 and ^###^*P* < 0.001 compared with their corresponding control group, Student’s *t*-test.

To determine nitric oxide (NO) bioavailability (basal NO release) in aortic segments, concentration-responses curves to Phe were performed in presence of L-NAME (10^−4^ M). L-NAME significantly increased contractile responses to Phe in both groups (Fig. 2B,C and Table 3) but to a higher extent in Chow mice as evidenced by the AUC calculated from the concentration-response curves to Phe in presence or absence of L-NAME (AUC_Chow_ = 356.2 ± 24.9 *vs* AUC_LF_ = 254.9 ± 22.7). Moreover, NO bioavailability estimated from the difference between the AUC in absence and in presence of L-NAME was also higher in Chow (206%) than in LF mice (163%) (Fig. 2D, see NO contribution in black).

### LF diet reduced vascular relaxant responses to Ach in the thoracic aorta and reduced NO contribution

**Figure 3:**
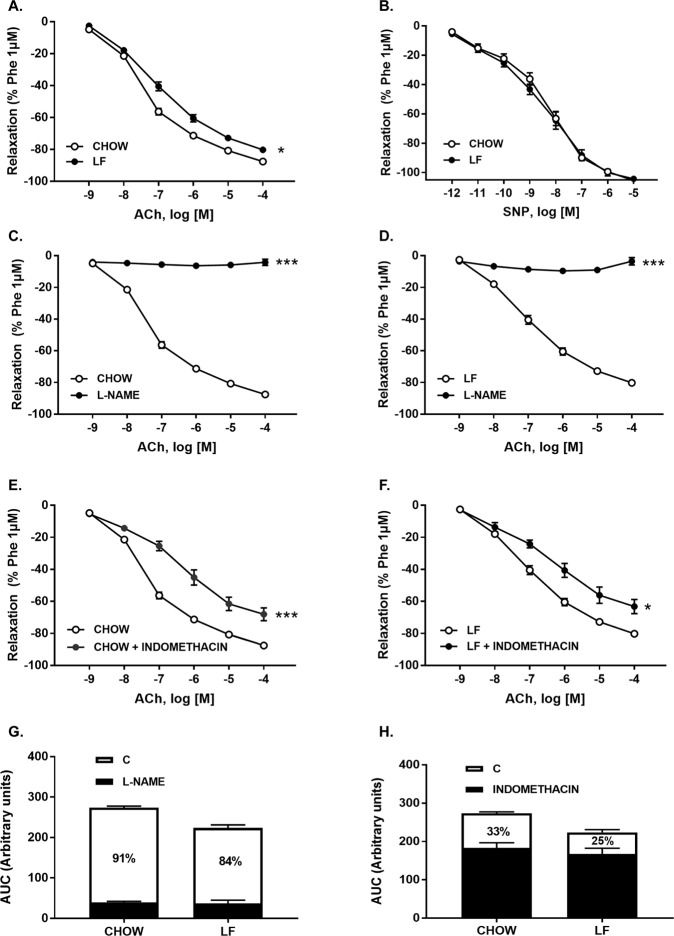
(**A**) Cumulative concentration-response curves to acetylcholine (10^−9^–10^−4^M) and (**B**) sodium nitroprusside (10^−12^–10^−5^M) in aortic segments from both Chow and LF animals. (**C**,**D**) Cumulative concentration-response curves to Ach (10^−9^–10^−4^M) in aortic segments from both Chow (**C**) and LF (**D**) animals pre-incubated or not with L-NAME (10^−4^ M). (**E**,**F**) Cumulative concentration-response curves to acetylcholine (10^−9^–10^−4^ M) in aortic segments from both Chow (**E**) and LF (**F**) animals pre-incubated or not with indomethacin (3 × 10^−6^M). The percentage of inhibition of relaxant responses elicited by L-NAME (**G**) or indomethacin (**H**) and shown in white bars indirectly reflects NO and PGI_2_ contribution, respectively. Data are expressed as mean ± SEM of 7–10 determinations per group. *P < 0.05 and ***P < 0.001 compared with the corresponding control group, Two-way analysis of variance (ANOVA) followed by Bonferroni’s comparison test (panels A–F), Student’s *t*-test (panels G,H).

The functional integrity of the endothelium was assessed with acetylcholine (Ach), a muscarinic receptor agonist and endothelial-dependent vasodilator. Concentration-response curves to Ach (10^−9^ to 10^−4^ M) induced a relaxation, that was significantly higher in mice fed with chow diet (Fig. 3A), as evidenced by E_max_ and pD_2_ values (Table 3). However, the sensitivity of aortic muscle to NO, assessed by concentration-response curves to the NO donor, sodium nitroprusside (SNP, 10^−12^–10^−5^ M) was similar in both groups (Fig. 3B).

The relative contribution of the main endothelial factors involved in the relaxation to Ach (NO and PGI_2_) was analyzed separately in each strain (Fig. 3C–F). To assess NO availability, that includes basal NO and NO released by Ach stimulation, aortic segments were preincubated with N_ω_-Nitro-L-arginine methyl ester hydrochloride (L-NAME; 10^−4^ M), a nitric oxide synthase inhibitor, before performing relaxing responses curves to Ach. L-NAME completely abolished the relaxation to Ach in both experimental groups (Fig. 3C,D). However, the relative contribution of NO to the relaxant response elicited by Ach, estimated from the difference between the AUC in absence and in presence of L-NAME, was significantly reduced by the LF diet (Chow = 91% vs LF = 84% and Fig. 3G, see NO contribution in white). We also analyzed the contribution of PGI_2_ to the relaxant response to Ach by preincubating the aortic rings with indomethacin (3 × 10^−6^ M), a cyclooxygenase inhibitor. Indomethacin significantly reduced Ach-induced relaxation in both experimental groups (Fig. 3E,F). However, no differences were detected in the net contribution of PGI_2_ as shown by the percentage of inhibition elicited by indomethacin (Chow = 33% vs LF = 25%; Fig. 3H; see PGI_2_ contribution in white).

### LF diet impaired relaxant responses to insulin in the thoracic aorta

**Figure 4:**
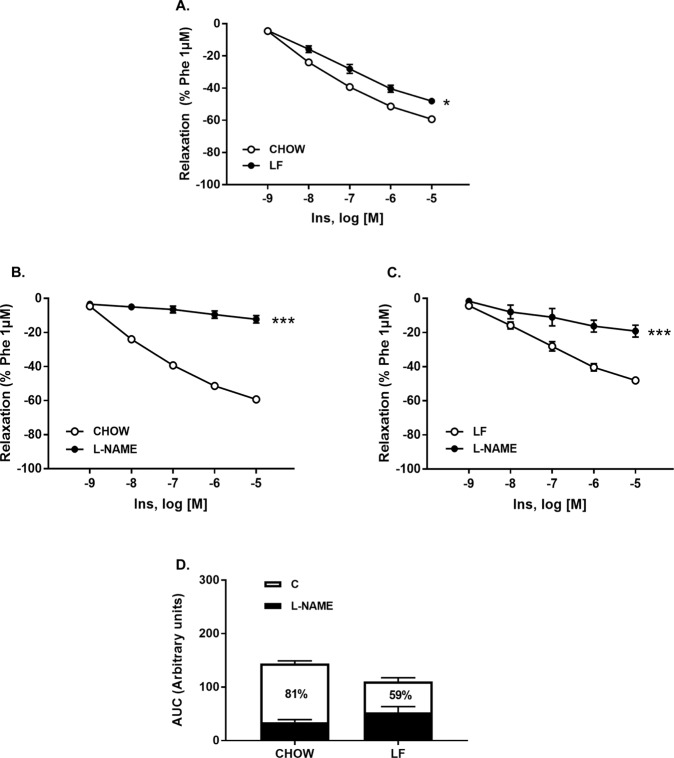
(**A**) Cumulative concentration-response curves to insulin (10^−9^–10^−5^ M) in aortic segments from both Chow and LF animals. (**B**,**C**) Cumulative concentration-response curves to insulin (10^−9^–10^−5^ M) in aortic segments from both Chow (**B**) and LF (**C**) animals pre-incubated or not with L-NAME (10^−4^ M). The percentage of inhibition of relaxant responses elicited by L-NAME (**D**) and shown in white bars indirectly reflects NO contribution. Data are expressed as mean ± SEM of 7–10 determinations per group. *P < 0.05 and ***P < 0.001 compared with the corresponding control group, Two-way analysis of variance (ANOVA) followed by Bonferroni’s comparison test (panels A–C), Student´s *t*-test (panel D).

Aortic insulin sensitivity was analyzed in aortic segments previously contracted with Phe (10^−6^ M). Relaxant responses to insulin (10^−9^ to 10^−5^ M) were significantly higher in Chow arteries compared with the LF group (Table 3 and Fig. 4A). In addition, although L-NAME completely abolished insulin relaxation in Chow animals, it was only partially reduced in the LF group (Fig. 4B,C). Therefore, and as evidenced by the percentage of inhibition elicited by L-NAME (Chow = 81% vs LF = 59%), the contribution of NO to insulin relaxation was significantly reduced by the LF diet (Fig. 4D; see NO contribution in black).

## Discussion

Choosing the proper control diet in metabolic and vascular studies is crucial for successful development of the research. The novel finding of this study is that there are substantial differences in the metabolism and vascular function between animals fed with two of the most common control diets: chow and purified LF diet. Animals fed with LF purified diet for 6 weeks exhibited impaired systemic glucose tolerance, endothelial function and vascular insulin sensitivity in C57BL6J mice even when both diets are considered isoenergetic.

We and others have already demonstrated the impact of hypercaloric diets based on either high fructose^8,9^, high sucrose^10,11^ or high fat content on both metabolic (i.e. insulin resistance and/or type II diabetes) and vascular alterations like endothelial dysfunction^3,5^. Nevertheless, it has been suggested that the nature of nutrients included in a diet and not only the energy provided might be crucial for the development of both metabolic and vascular alterations.

It is currently well known that purified LF and standard chow diets composition differs notably while being considered isoenergetic. One of the major differences between those diets is the percentage of carbohydrates. Indeed, whereas chow diets are rich in fiber and mainly contain complex carbohydrates, purified LF diets are mainly composed of easy metabolizable carbohydrates (sucrose, dextrin, etc.) and only contain non-soluble fiber (cellulose)^6^.

Our results demonstrate that after only 6 weeks we observed changes in the metabolic profile due to the differential nutritional composition. The 20% increase in the energy content observed in the LF diet was reflected in an impact on the circulating levels of insulin and lipids. The hyperinsulinemia, together with elevated HOMA-IR and the results obtained from the GTT are reflective of defective insulin signaling. Regarding the plasma lipid profile, even when both diets supply a similar amount of fatty acids, LF animals exhibit an increase esterification into TG, according to the concomitant elevation of TG and reduction of NEFA. Cholesterol content in the chow diet was null, compared to the 18 ppm of the LF, which may account for the 25% increase in plasma cholesterol detected in the LF-fed animals. To confirm this status, a GTT was performed, revealing a low glucose clearance in the LF diet-fed animals together with an increase of insulin levels 15 min after glucose injection. These results are in accordance with previous studies that showed a similar trend, even after only 3 weeks of feeding^12^. Nevertheless, to further deep into the whole-body insulin action, an insulin tolerance test would be recommended.

One of the major results of this study is that endothelial function was impaired by LF diet, as shown by the reduction in aortic relaxation induced by Ach. Nonetheless, this reduction of E_max_ of about a 7% is moderate as compared to the effects induced by a very HFD (Emax reduction of more than 30%)^3^. Interestingly, L-NAME preincubation induced a higher increase in contractile responses to Phe in Chow mice than in LF animals, demonstrating a significant reduction of basal NO availability in the latter group. In addition, and since the inhibition of relaxation to Ach in presence of L-NAME was also smaller in LF mice, total NO availability, comprising both basal NO and NO released in response to Ach, appeared to be reduced by LF diet. However, the reduction in NO contribution induced by the LF diet is also smaller to the one induced by HFD^3^. Similarly, we observed a reduction in insulin-induced relaxation in LF mice of at about a 10% as compared with Chow animals. However, studies performed in mice fed a very HFD exhibited a reduction of insulin relaxation of about 50%^3^. In light of these results, we can state that purified diets induce an impairment of endothelial function and vascular insulin sensitivity due to a reduced contribution of NO as it has been described with HFDs. However, this effect is very moderate compared to what was observed by hypercaloric HFDs.

We also evaluated if LF diet could modify the contribution of prostanoids, other major vasodilators involved in endothelial relaxation. In this regard, and since preincubation with indomethacin elicited the same effect in both LF and Chow fed animals, we can discard a differential contribution of prostanoids to aortic relaxation in LF mice. Intriguingly, a study performed in rats fed with a HFD revealed no changes in prostanoids contribution to endothelial relaxation^4^. Consequently, those data suggest that vascular alterations associated with intake of both LF and HF diets are mainly due to alterations in NO production.

Another important difference between purified LF and chow diets is the type of fiber provided. Indeed, while fiber contained in chow diets often includes a mixture of cellulose, hemicellulose, lignin and pectin LF diets only contains cellulose, a non-soluble type of fiber^13^. Interestingly, an original study performed in Japanese patients with type 2 diabetes demonstrated that a fiber-rich diet based on brown rice for 8 weeks effectively improved endothelial function^14^. In addition, it seems that soluble fiber, absent in LF diets, might prevent obesity and metabolic syndrome-related inflammation because of its beneficial role on the microbiota^15^. In fact, the addition of these type of soluble fibers to purified diets has been proved to exert beneficial effects by diminishing adiposity and improving glucose tolerance^13,16^. Similarly, a preclinical study has shown a beneficial effect of inulin-type fructans (a kind of soluble fiber) supplementation on endothelial dysfunction in both mesenteric and carotid arteries of n-3 PUFA-depleted Apoe^−/−^ mice through the activation of the nitric oxide (NO) synthase/NO pathway^17^. Since, we have detected a reduction of NO availability in mice fed with a LF diet that only contains insoluble fiber (cellulose) but no kind of soluble fiber, we could hypothesize that the lack of soluble fiber in LF diets could also contribute to impairing endothelial function. Nevertheless, further experiments would be required to clarify this point.

Other important nutrients that are contained in smaller quantities in purified LF diets are some vitamins like vitamin A, D and E. The protective role of vitamins to decrease cardiovascular and metabolic risk is still a matter of debate. The most recent meta-analysis demonstrates that a higher vitamin C intake, and higher circulating levels of vitamin C, E and β-carotene are associated with a lower risk of cardiovascular disease mortality^18^. In this regard, vitamin A deficiency has shown to induce structural alterations in the rat aorta that might be associated with oxidative stress and inflammation^19^. Similarly, it is currently well accepted that vitamin D deficiency might account for the development of inflammation-linked endothelial dysfunction^20^. Nevertheless, there are some controversial results around this matter and some authors state that vitamin D supplementation have not been demonstrated to improve endothelial function in overweight/obese non-hypertensive individuals^21^. In contrast, a protective role of vitamin E supplementation on vascular endothelial function has been described, especially in hypertensive patients^22^.

Finally, there are also qualitative differences regarding the type of fatty acids contained in purified LF versus chow diets, being especially different in the amount of linoleic acid, two-fold higher in the chow than in the LF diet. Interestingly, an inverse association between linoleic acid intake and the risk of type 2 diabetes has been recently reported, especially when the reduced amount of this fatty acid is replaced by saturated fatty acids, *trans* fats, or carbohydrates^23^, as it is the case in purified LF diets.

In conclusion, this study demonstrates that while purified LF diets do not induce an increase of body weight compared to the chow diet, it induces a moderate impairment of glucose tolerance, aortic endothelial function and aortic insulin sensitivity. Those effects could be attributed to an increased supply of simple carbohydrates together with a lack of soluble fiber, a reduction of vitamin supply and a reduction of the amount of PUFA like linoleic acid.

## Materials and Methods

### Animals

All experiments were performed in C57BL/6 J mice, from Charles River strains and inbred at Universidad San Pablo-CEU animal facilities and housed under controlled light (12:12 h light-dark cycles), temperature (22–24 °C) and relative humidity (44–55%) with standard food and water ad libitum. Four to five-week-old male mice, weighing 16–18 g, were divided into two groups with similar average body weight (BW; n = 5 mice/cage) and fed either with a standard chow diet (Chow; n = 10) or a low-fat diet (LF; n = 7) for 6 weeks. Both BW and food intake were monitored weekly. Energy consumption was calculated from food intake values and expressed as Kcal/day/mice. Caloric efficiency was calculated from BW increase/Kcal intake per animal per week and expressed as g/Kcal. On the last day, mice were weighed and euthanized by decapitation at 9 am. Thoracic aorta was immediately removed and used for vascular function studies. The liver, heart, perirenal (PR-AT), subcutaneous (SC-AT) periaortic (PA-AT) and mesenteric adipose tissue (Mes-AT) were dissected, weighed, and their weight was normalized by tibia length. Blood was collected in chilled EDTA-coated polypropylene tubes, centrifuged a 3000 rpm for 20 min to obtain the serum, which was stored at −80 °C until biochemical analysis. All experimental procedures were performed in accordance with the European Union Laboratory Animal Care Rules (86/609/ECC directive) and were approved by the Animal Research Committee of San Pablo CEU University (PROEX 061/16).

### Diets

Chow diet (Teklad Rodent Diet 2918 from Envigo (U.S.A)) provides 3.1 kcal/g (0.56 kcal/g from fat, 1.8 kcal/g from carbohydrates and 0.75 kcal/g from protein). LF diet (D12450B from Test Diet (UK)) provides 3.76 kcal/g (0.38 kcal/g from fat, 2.7 kcal/g from carbohydrates and 0.68 kcal/g from protein). Both diets, chow and LF, are considered isoenergetic (3–4 kcal/g) but they differ in protein, carbohydrates, total fat and vitamins content. In addition, the source of energy from carbohydrates and fat is also different. Details of diet composition (provided by the manufacturers) appear summarized in Table 1.

### Analysis of fatty acids, carbohydrates and amino acids presents in the diets by ^1^H HR-MAS NMR

Samples was examined using high resolution magic angle spinning (HR-MAS) operating at 4 °C to minimize sample degradation and placed within a 50 μl zirconium oxide rotor with cylindrical insert and spun at 5000 Hz spinning rate. 1H-NMR spectroscopy was performed at 500.13 MHz using a Bruker AVIII500 HD spectrometer 11.7 T. Shimming and NMR preparation time was tried to keep to a minimum, but throughout this the NMR analysis was chilled to 4 °C to minimize metabolic changes. Under such conditions no noticeable degradation was observed during acquisition.

Standard solvent suppressed spectra were acquired into 32 k data points, averaged over 128 acquisitions, total acquisition ∼11 min using a standard Bruker sequence (noesypr1d) with a relaxation delay of 2 s and a mixing time of 150 ms. A spectral with of 6009.62 Hz was used. All spectra were processed using TOPSPIN software, version 3.5 (Bruker Rheinstetten, Germany). Prior to Fourier transformation, the FIDs were multiplied by an exponential weight function corresponding to a line broadening of 0.3 Hz. Spectra were phased, baseline-corrected and referenced to the sodium (3-trimethylsilyl)-2,2,3,3-tetradeuteriopropionate singlet (TSP) at δ 0ppm.

^1^H, ^13^C 2D experiments was performed to carry out the component assignments. HSQC experiments were registered with the following parameters: 6009 Hz and 22 kHz spectral widths in the 1 H and 13 C dimensions, respectively, 2 k data points in f2 and 192 increments in f1. Zero filling in f1 and unshifted squared sinusoidal window function in both dimensions were applied before Fourier transformation.

### Plasmatic determinations

Biochemical determinations were analyzed in plasma samples obtained under fasting conditions (12 h). Insulin was determined by means of a specific enzyme immunoassay kit for mouse insulin (Mercodia, Denmark) (2.2% intra-assay variation, 4.9% inter-assay variation). Glucose (glucose Trinder Method, Roche Applied Science, Barcelona, Spain), triglycerides (glycerol phosphate oxidase method, Biolabo, Maizy, France), non-esterified fatty acids (Acyl-CoA oxidase method, Wako, Bioproducts, Germany) and cholesterol (cholesterol oxidase - peroxidase method, Spinreact, Barcelona, Spain) were measured by colorimetric methods.

### Intraperitoneal glucose tolerance test (GTT) and homeostasis model assessment of insulin resistance (HOMA-IR)

A week before being euthanized, mice were fasted for 6 h before the glucose load (bolus of 1 g/kg i.p. at time 0). Glucose levels were measured in blood samples, drawn from the tail vein of conscious mice, at 0, 15, 30, 45, 60, 90, and 120 min after injection, with an Accu-Chek Aviva glucometer (Roche Diagnostics, Germany). Insulin levels were also determined in plasma samples obtained 15 min after glucose injection. HOMA-IR index was calculated with the following formula: [fasting blood glucose (mmol/L) × fasting plasma insulin (µU/mL)]/22.5^24^.

### Functional studies in the thoracic aorta artery

Thoracic aortas were carefully dissected and placed in cold physiological Krebs buffer solution (115 mM NaCl, 4.6 mM KCl, 2.5 mM CaCl_2_, 25 mM NaHCO_3_, 1.2 mM KH_2_PO_4_, 1.2 mM MgSO_4_, 0.01 mM EDTA and 11.1 mM glucose), cleaned of perivascular adipose tissue and cut into rings of 2–3-mm length. Aortic rings were then suspended on two intraluminal parallel wires connected to a force transducer and introduced to an organ bath containing oxygenated Krebs at 37 °C and 95% O_2_-5% CO_2_ (pH 7.4). Isometric tension was recorded in a Power Lab system (ADInstruments, Oxford, UK). Arterial rings were given an optimal resting tension of 1 g, which was readjusted every 10 min during a 40 min equilibration period. Before starting the experiment, arterial contractility was checked using a potassium solution (KCl, 75 mM). Cumulative concentration-response curves to phenylephrine (Phe, 10^−8^–10^−6^ M), acetylcholine (Ach, 10^−9^–10^−4^ M), sodium nitroprusside (SNP, 10^−12^–10^−5^ M) and insulin (10^−10^–10^−5^ M) were performed. Aortic rings were pre-contracted with a submaximal concentration of Phe (10^−6^ M) prior to Ach, SNP and insulin relaxation curves. NG-nitro-L-arginine methyl ester (L-NAME, 10^−4^ M) and indomethacin (3 × 10^−6^ M) were preincubated for 20 min. All the reagents were provided by Sigma-Aldrich (USA).

### Data analyses

Contractile responses to Phe are expressed as the percentage of contractions elicited by 75 mM KCl. Relaxations are expressed as the percentage of the previous contraction elicited by Phe. The maximal response (E_max_ values) and the potency (pD_2_ values) were calculated by nonlinear regression analyses of each individual concentration-response curve. The area under the curve (AUC) was calculated from each individual concentration-response curve plot. Results are expressed as mean ± SEM and n denotes the number of animals used in each experiment. Comparison between groups were made by performing a two-way analysis of variance (ANOVA) or Student’s *t*-test as appropriate using GraphPad Prism 07 (San Diego, CA, USA). Post-hoc comparisons were carried out with the Bonferroni’s test. Statistical significance was set at *P* < 0.05.
